# Increased RET Activity Coupled with a Reduction in the *RET* Gene Dosage Causes Intestinal Aganglionosis in Mice

**DOI:** 10.1523/ENEURO.0534-20.2021

**Published:** 2021-05-28

**Authors:** Mitsumasa Okamoto, Toshihiro Uesaka, Keisuke Ito, Hideki Enomoto

**Affiliations:** Division for Neural Differentiation and Regeneration, Department of Physiology and Cell Biology, Kobe University Graduate School of Medicine, Kobe 650-0017, Japan

**Keywords:** cell signaling, enteric nervous system, Hirschsprung’s disease, neuronal differentiation, RET

## Abstract

Mutations of the gene encoding the *RET* tyrosine kinase causes Hirschsprung’s disease (HSCR) and medullary thyroid carcinoma (MTC). Current consensus holds that HSCR and MTC are induced by inactivating and activating RET mutations, respectively. However, it remains unknown whether activating mutations in the *RET* gene have adverse effects on ENS development *in vivo*. We addressed this issue by examining mice engineered to express RET51(C618F), an activating mutation identified in MTC patients. Although *Ret^51(C618F)/51(C618F)^*mice displayed hyperganglionosis of the ENS, *Ret^51(C618F)/-^* mice exhibited severe intestinal aganglionosis because of premature neuronal differentiation. Reduced levels of glial cell-derived neurotrophic factor (GDNF), a RET-activating neurotrophic factor, ameliorated the ENS phenotype of *Ret^51(C618F)/-^* mice, demonstrating that GDNF-mediated activation of RET51(C618F) is responsible for severe aganglionic phenotype. The *RET51(C618F)* allele showed genetic interaction with *Ednrb* gene, one of modifier genes for HSCR. These data reveal that proliferation and differentiation of ENS precursors are exquisitely controlled by both the activation levels and total dose of RET. Increased RET activity coupled with a decreased gene dosage can cause intestinal aganglionosis, a finding that provides novel insight into HSCR pathogenesis.

## Significance Statement

Mutations of the *RET* gene have been identified in Hirschsprung’s disease (HSCR) and neuroendocrine tumors (NET). It has been thought that HSCR and NET are caused by inactivating and activating mutations of the RET gene, respectively. However, little is known about whether enhanced RET activity exerts any roles in the pathogenesis of HSCR. We show that mice carrying an activating mutation in the *Ret* gene display intestinal aganglionosis when the *Ret* gene dosage is halved. The aganglionosis phenotype is caused by premature neuronal differentiation and impaired migration of ENS precursors. These findings raise the possibility that RET-activating mutations can cause HSCR when associated with a reduction in the dosage or expression of the *RET* gene.

## Introduction

RET is a receptor tyrosine kinase that serves as a signaling receptor for the glial cell-derived neurotrophic factor (GDNF) family ligands (GFLs; Baloh et al., 2000; Airaksinen and Saarma, 2002). Binding of GFLs to their cognate GFRα receptors induces dimerization and subsequent autophosphorylation of RET, culminating in the activation of downstream intracellular signaling. RET is expressed in a wide variety of neural crest (NC)-derived cell types and endoderm-derived thyroid C cells. In humans, mutations in the *RET* gene are associated with the pathogenesis of various forms of diseases that include Hirschsprung’s disease (HSCR) and medullary thyroid carcinoma (MTC). Hereditary MTCs occurs in multiple endocrine neoplasia (MEN)2, which is further subcategorized into MEN2A, MEN2B, and familial MTC (FMTC) based on the phenotype such as pheochromocytoma, hyperparathyroidism and/or other developmental anomalies (infertility, marfanoid habitus, etc.; Wells et al., 2013; Tomuschat and Puri, 2015).

HSCR is characterized by the congenital loss of enteric ganglia in the distal portion of the gut (intestinal aganglionosis). Genetic studies have revealed that HSCR is a multifactorial disease that involves mutations in multiple genes for its pathogenesis and exhibits complex patterns of inheritance. To date, mutations have been identified in as many as 17 genes (Tilghman et al., 2019), among which the RET gene is most frequently mutated (Amiel et al., 2008). HSCR-associated RET mutations have been identified throughout the *RET* genome, affecting both coding and non-coding regions. Coding mutations account for only 50% of familial and 15% of sporadic cases of HSCR (Edery et al., 1994; Romeo et al., 1994). Meanwhile, some non-coding variants that potentially affect the enhancer activity of the RET gene are considered necessary, albeit not sufficient, mutations in isolated HSCR cases (Kapoor et al., 2015). Thus, HSCR is a complex genetic trait in which reduced RET expression confers susceptibility to the disease.

In contrast to HSCR, MEN2 displays rather simple genetic features. Most of those mutations affects a restricted cysteine residue in the extracellular domain of RET, converting it to arginine, tyrosine or phenylalanine. These amino acid conversions disrupt intra-molecular cysteine bonding and causes aberrant intermolecular bonding and successive auto-phosphorylation of RET, leading to its aberrant and ligand-independent activation. Those RET mutations are likely sufficient for the development of tumors because familial MEN2 cases demonstrate autosomal dominant inheritance (Margraf et al., 2009).

Together, HSCR-associated and MTC-associated RET mutations display distinct features, and, although the whole spectrum of biological effects by those RET mutations have not been fully elucidated, current consensus holds that HSCR is caused by inactivating mutations of the *RET* gene whereas MTC is induced by activating mutations of RET (Hansford and Mulligan, 2000).

Although previous studies support that inactivation or downregulation of RET signaling leads to HSCR-like intestinal aganglionosis in mice, it has not been clear whether activating mutations of RET have adverse effects on ENS development (Amiel et al., 2008). To address this issue, we examined the development of the ENS in mice engineered to express RET C618F, one of the MTC-associated RET-activating mutants, under the endogenous *Ret* promoter (Okamoto et al., 2019). Biochemical studies revealed that RET C618F displays slightly higher RET basal phosphorylation than normal, but still requires GDNF for its full activation (Okamoto et al., 2019). Thus, RET C618F mutant mice are an ideal platform to understand how the ENS develops when the activity of RET is slightly elevated. We found that, in mice carrying the RET C618F mutation, the ENS phenotype changed dramatically from hyperganglionosis to aganglionosis when the Ret gene dosage was changed from two copies to one copy. Premature neuronal differentiation of ENS precursors contributed to the aganglionosis phenotype. Our findings reveal a novel mechanism of HSCR pathogenesis that is Ret-activating mutations can cause HSCR when the Ret gene dosage is reduced.

## Materials and Methods

### Mice

The generation and characterization of *Ret51* and *Ret51*(C618F) mice have been described previously (Okamoto et al., 2019). We obtained *Ret^GFP^* (a kind gift from J. Milbrandt, Washington University School of Medicine; Jain et al., 2006), *Gdnf^+/−^* (a kind gift from V. Pachnis The Francis Crick Institute, London, UK; Moore et al., 1996), and *Ednrb^flex3^* mice (a kind gift from M. L. Epstein, University of Wisconsin-Madison; Druckenbrod et al., 2008). *Ednrb^+/−^* mice were obtained by crossing *Ednrb^flex3^*mice to *Actb::Cre* mice (stock #019099; The Jackson Laboratory; Lewandoski et al., 1997).

Mice were bred and maintained at the Institute of Experimental Animal Research of Kobe University Graduate School of Medicine under specific pathogen-free conditions and all animal experiments were performed according to the Kobe University Animal Experimentation Regulations.

#### Whole-mount immunostaining

Dissected gut from embryos or postnatal day (P)0 pups were fixed with 4% paraformaldehyde (PFA) in PBS containing 10 mm phosphate buffer, pH 7.4, 137 mm sodium chloride, and 2.7 mm potassium chloride overnight at 4°C and incubated in 1% Triton X-100 in PBS for 30 min at room temperature. After fixation and permeabilization, the preparations were incubated in 0.1 m glycine in PBS for 2–6 h and processed for immunohistochemistry. For the preparations from P0 pups, blocking solution contains 5% skim milk, 5% DMSO, 1% Tween 20 in PBS. The following antibodies were used: guinea pig anti-Phox2b (1:1000, home-made, raised against the C-terminal region of Phox2b (RRID:AB_2313690; Pattyn et al., 1997), goat anti-Sox10 (1:300, catalog #sc-17342, Santa Cruz Biotechnology Inc., RRID: AB_2195374), rabbit anti-PGP9.5 (1:1000, catalog #RA-95 101, Ultra Clone, RRID: AB_2313685), rabbit anti-phospho-ERK1/2 (1:500, catalog #9101, Cell Signaling Technology, RRID: AB_331646), and chicken anti-GFP (1:1000, catalog #GFP-1020, Aves Laboratories, RRID: AB_10000240). We used the following secondary antibodies (Biotium): CF488A donkey anti-rabbit IgG (catalog #20015, RRID: AB_10559669), CF488A donkey anti-chicken IgY (catalog #20166, RRID: AB_10854387), CF568 donkey anti-guinea-pig IgG (catalog #20377), CF568 donkey anti-goat IgG (catalog #20106, RRID: AB_10559672), CF568 donkey anti-rabbit IgG (catalog #20098, RRID: AB_10557118), and CF640T donkey anti-goat IgG (catalog #20179, RRID: AB_10853145).

#### Whole-mount gut 5-ethynyl-2'-deoxyuridine (EdU) assays

Embryonic day (E)12.5 or E14.5 pregnant females were injected intraperitoneally with EdU (50 μg/g body weight). Two hours after injection, dissection of gut was followed by fixation and permeabilization in the same fashion as whole-mount immunostaining. The preparations were washed twice for 3 min with 3% BSA in PBS at room temperature. For EdU assays (Click_-_iT Plus EdU Imaging kit, Invitrogen), the reaction cocktail (reaction buffer, CuSO_4_, Alexa Flour 594 azide and buffer additive as per manufacture’s protocol) was added for 30 min followed by rinsing twice for 3 min with 3% BSA in PBS in the dark at the room temperature. After EdU labeling, whole-mount immunostaining was performed as described above.

#### Cell counts

Phox2b^+^ neurons were counted on ten sections per investigated region of the gut at P0. Phox2b^+^and EdU^+^Phox2b^+^ ENS precursors were counted in a minimum of five areas at even intervals of the midgut longitudinally (0.025 mm^2^ each) and the rate of ENS precursor proliferation was determined in animals for each genotype.

#### Experimental design and statistical analysis

Images were carefully selected to show the average effect obtained for each experimental condition. All descriptive statistics are presented as means ± SEM. Normality of the data were tested with Levene’s test (SPSS II Statistics software; SPSS Inc.), and differences were subsequently assessed using unpaired *t* test. If assumptions for a parametric test were not met (Levene’s test, *p* < 0.05), unpaired *t* test with Welch correction was used. Statistical analyses for enteric neuron numbers were performed using one-way analysis of variance (ANOVA), followed by pairwise comparisons (Tukey’s *post hoc* test) where appropriate. GraphPad Prism 5 software (GraphPad Software Inc.) was used to conduct unpaired *t* test with Welch correction, one-way ANOVA with Tukey test and χ^2^ test. Animals of both sexes were used. No methods were used for sample size determination.

## Results

### RET(C618F) enhances proliferation of ENS precursors and causes intestinal hyperganglionosis

**Figure 1.**
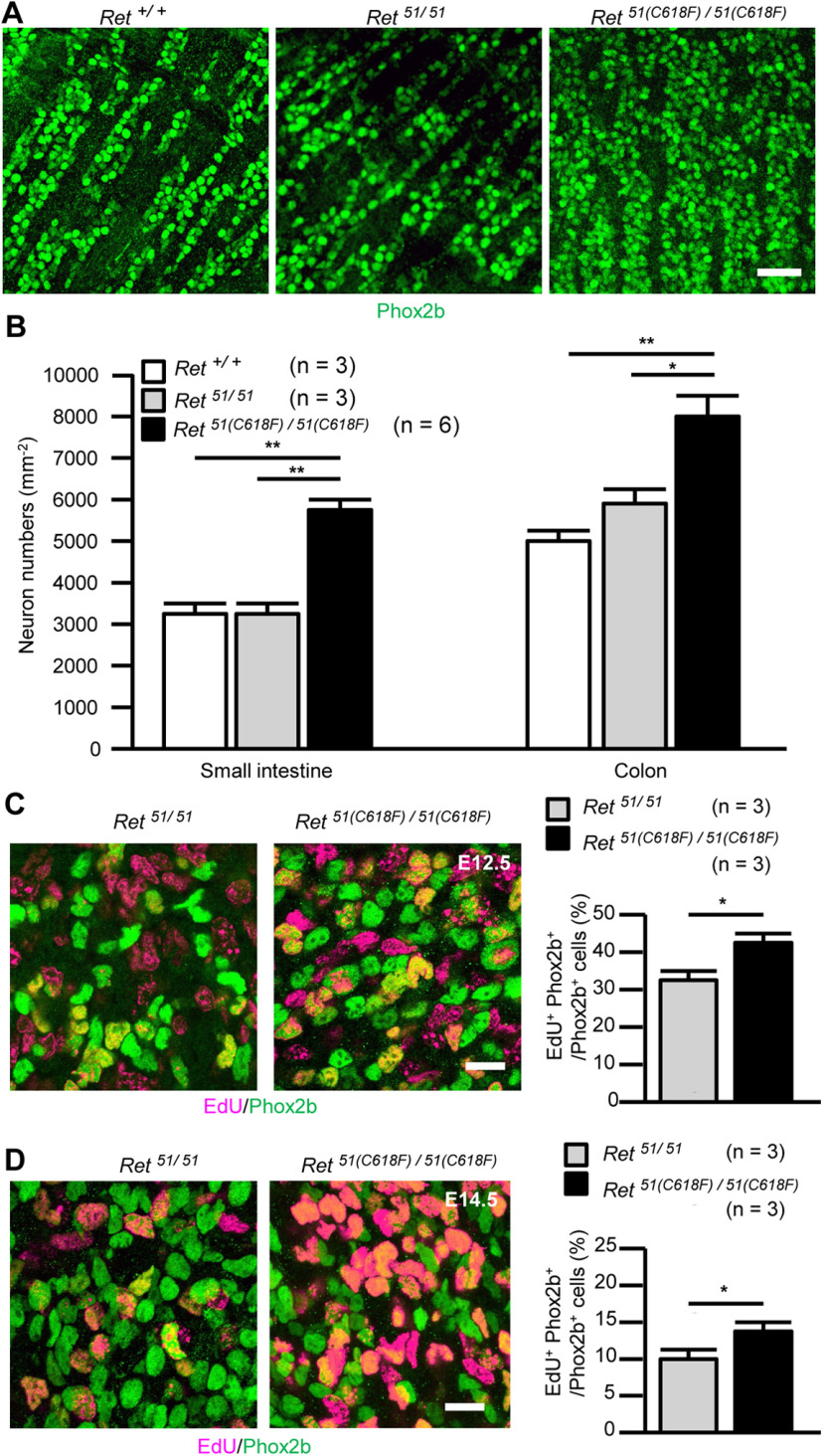
*Ret^51(C618F)/51(C618F)^* mice display hypertrophy of enteric ganglia. ***A***, Whole-mount Phox2b staining (green) of enteric neurons in the myenteric plexus of the small intestine from P0 *Ret^+/+^*, *Ret^51/51^*, and *Ret^51(C618F)/51(C618F)^* mice. ***B***, Quantification of Phox2b^+^ enteric neuron numbers in the small intestine and the colon from P0 *Ret^+/+^* (*n* = 3), *Ret^51/51^* (*n* = 3), and *Ret^51(C618F)/51(C618F)^* (*n* = 6) mice; *p =* *0.001, **p <0.0001, one-way ANOVA with Tukey’s *post hoc* test. ***C***, ***D***, Detection of EdU (magenta) incorporated into Phox2b^+^ ENS precursors (green) in the midgut from E12.5 (***C***) and E14.5 (***D***) *Ret^51/51^* and *Ret^51(C618F)/51(C618F)^* fetuses. The gut was labeled by a 2-h pulse of EdU. The graphs (right panels) display the rate of ENS precursor proliferation at E12.5 and 14.5 *Ret^51/51^* (*n* = 3) and *Ret^51(C618F)/51(C618F)^* (*n* = 3) fetuses; *p <0.05, unpaired *t* test. Scale bars: 50 μm (***A***) and 20 μm (***C***, ***D***).

To understand the biological impact of enhanced RET signaling on ENS development, we examined mice expressing RET(C618F), a MEN2-associated RET-activating mutant. Previous studies revealed that, among MEN2-associated RET mutants, those affecting RET(C618) residues display moderate to low transforming activity *in vitro* (Carlomagno et al., 1997; Ito et al., 1997). Our biochemical characterization indicated that RET(C618F) displays slightly higher basal phosphorylation than normal and requires GDNF for its full phosphorylation (Okamoto et al., 2019). Because RET(C618F) retains GDNF-responsiveness and exhibits moderate activation of RET-signaling, RET(C618F) is an ideal RET mutant to examine the effect of slight RET-signaling enhancement on ENS development. Since mice expressing RET(C618F) were engineered to express RET51(long isoform) cDNA carrying a C618F mutation by the endogenous *Ret* promoter, the mutant allele is hereafter referred to as 51(C618F). As a control, mice expressing wild-type *RET51* cDNA were used (the allele referred to as 51). *Ret^51(C618F)/51(C618F^*^)^ mice were born apparently normally at an expected Mendelian ratio but all died of unknown causes within 24 h after birth (Okamoto et al., 2019). In newborn (P0) *Ret^51(C618F)/51(C618F^*^)^ mice, histologic analysis of the gut revealed that the density of enteric neurons in the myenteric layer of the small intestine appeared higher in *Ret^51(C618F)/51(C618F^*^)^ mice than *Ret^51/51^* mice (control; Fig. 1*A*). Neuronal count confirmed a significant increase in the numbers of myenteric neurons in both small intestine and colon of *Ret^51(C618F)/51(C618F^*^)^ mice (*p *<* *0.0001; Fig. 1*B*).

We investigated proliferation of ENS precursors by anti-Phox2b staining (which detects almost all ENS precursors during mid-gestation) combined with EdU labeling at E12.5 (a period of ENS precursor migration) and E14.5 (a period when ENS precursor migration is completed). This analysis revealed an increase in double-positive cell populations in *Ret^51(C618F)/51(C618F^*^)^ embryos as compared with *Ret^51/51^*embryos in both of these developmental periods [*Ret^51(C618F)/51(C618F^*^)^ vs *Ret^51/51^* in the midgut; 43.3 ± 2.9% vs 31.7 ± 0.6% (*p *=* *0.019) at E12.5 and 13.9 ± 2.1% vs 10.2 ± 0.9% (*p *=* *0.046) at E14.5, respectively; Fig. 1*C*,*D*]. Thus, the increase in enteric neuron numbers in newborn *Ret^51(C618F)/51(C618F^*^)^ mice is attributed at least in part to enhanced proliferation of ENS progenitors.

**Figure 2.**
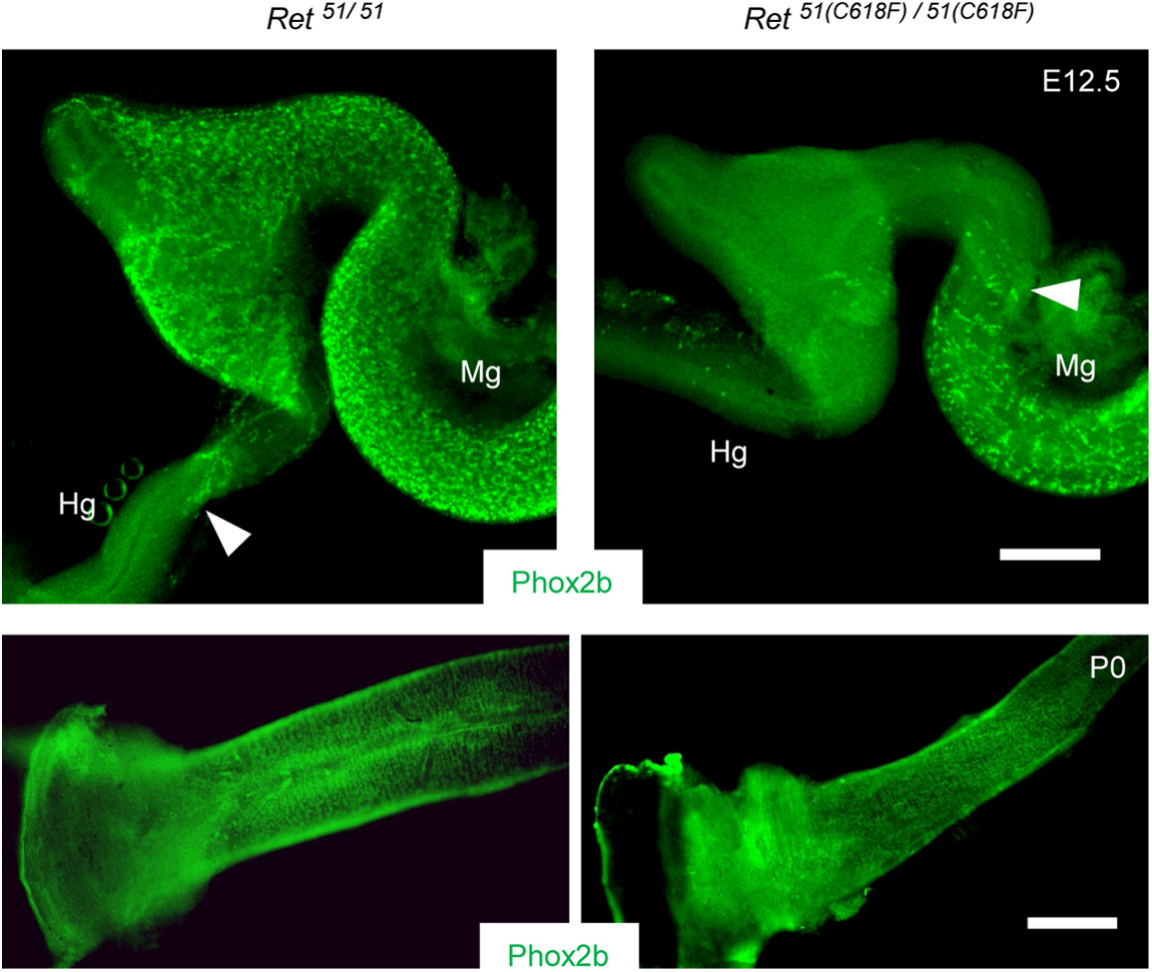
*Ret^51(C618F)/51(C618F)^* mice exhibit complete gut colonization by ENS precursors. Phox2b-labeled ENS precursors and neurons (green) in the developing gut in *Ret^51/51^* and *Ret^51(C618F)/51(C618F)^* mice at E12.5 (upper panels) and P0 (lower panels). Migration of ENS precursors is slightly delayed at E12.5, but gut colonization by them is completed at P0. Arrowheads depict the front of the migrating ENS precursors. Hg, hindgut; Mg, midgut. Scale bars: 250 μm (upper panels) and 500 μm (lower panels).

Previous studies suggested that reduced RET signaling impairs ENS migration (Young et al., 2001; Natarajan et al., 2002; Uesaka et al., 2008) and that proliferation of ENS precursors is a major driving force for ENS migration (Landman et al., 2007). We therefore examined the migration of ENS precursors in *Ret^51(C618F)/51(C618F^*^)^ embryos. Unexpectedly, the migratory wavefront of ENS precursors was always slightly delayed in *Ret^51(C618F)/51(C618F^*^)^ embryos as compared with control embryos at E12.5 (Fig. 2, upper panel). However, this delay was only transient and compensated for before birth. The ENS was fully developed in all of *Ret^51(C618F)/51(C618F^*^)^ neonates (Fig. 2, lower panel).

In adult *Ret^51(C618F)/+^* mice, we detected focal hyperplasia of thyroid C cells (Okamoto et al., 2019), a precancerous condition that leads to MTC (Wolfe et al., 1973). Together, these results indicate that, consistent with its enhanced activity *in vitro*, RET51(C618F) confers gain-of-function effects on development of the ENS and thyroid C cells.

### *Ret^51(C618F)/-^* mice display intestinal aganglionosis

**Figure 3.**
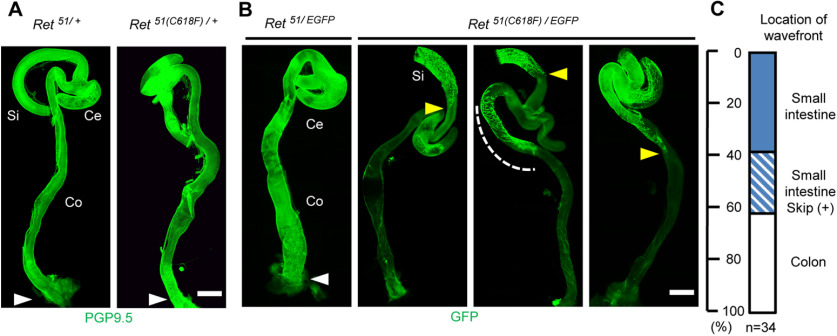
*Ret^51(C618F)/–^* mice exhibit intestinal aganglionosis. ***A***, ***B***, Whole-mount images of the enteric neurons stained with anti-PGP-9.5 (***A***) or labeled by GFP (***B***) in P0 *Ret^51/+^*and *Ret^51(C618F)/+^*, *Ret^51/EGFP^*, and *Ret^51(C618F)/EGFP^*gut. Complete gut colonization by ENS cells was seen in *Ret^51/+^*, *Ret^51(C618F)/+^*, and *Ret^51/EGFP^* mice (white arrowheads), while *Ret^51(C618F)/EGFP^* mice exhibited disrupted colonization of the gut by ENCCs. The wavefront (yellow arrowheads) was defined as the most caudal continuous strands of EGFP^+^ cells. Some *Ret^51(C618F)/EGFP^* mice show skip segment aganglionosis where small regions of the colon contain enteric ganglia (white dotted region). ***C***, The proportion of three types of aganglionic phenotype (small intestinal, skip segment, and colonic aganglionosis). Ce, cecum; Co, colon; Si, small intestine. Scale bars: 1 mm (***A***, ***B***).

**Figure 4.**
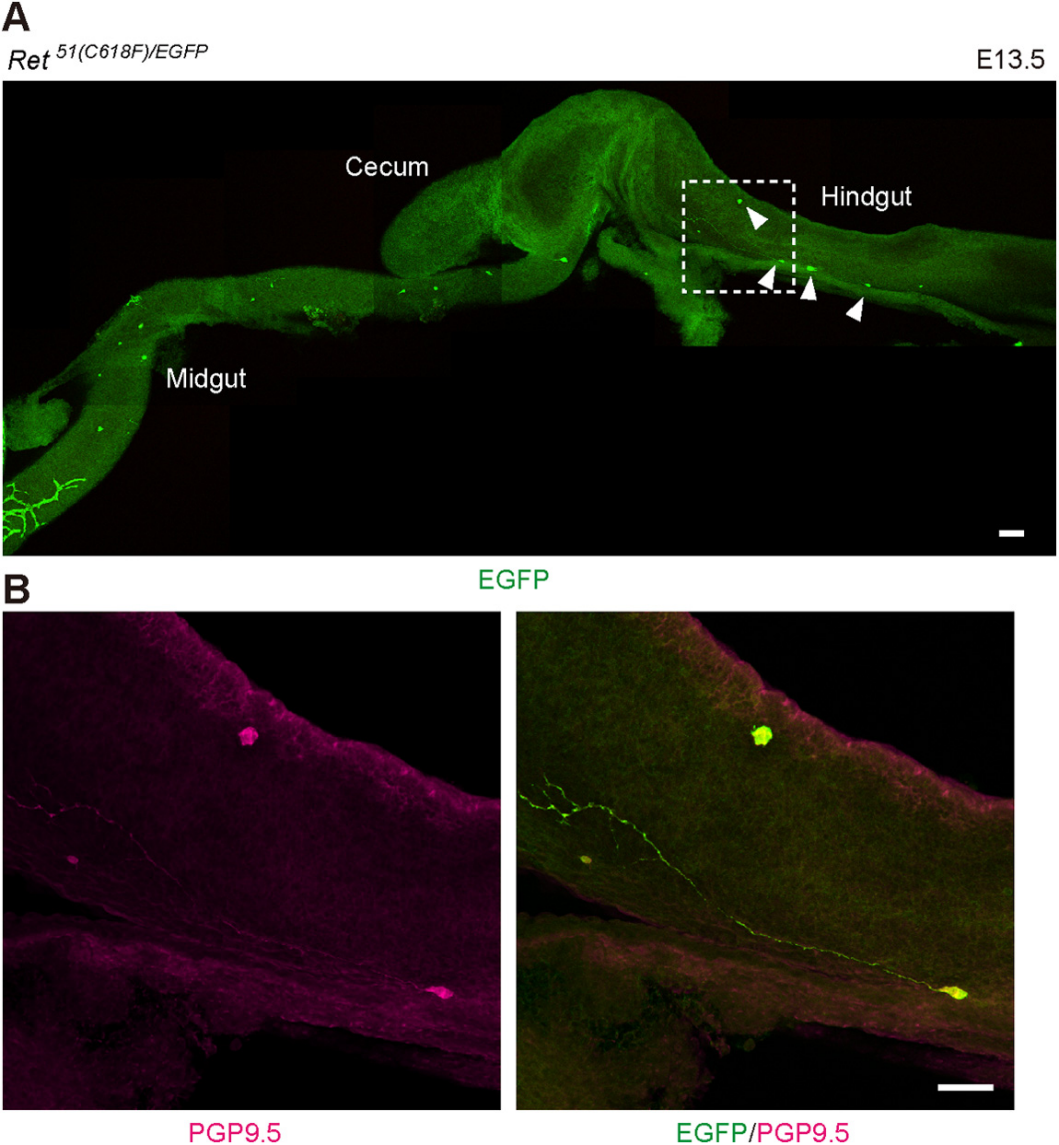
Detection of a few enteric neurons in the hindgut of *Ret^51(C618F)/EGFP^* embryos. Whole-mount preparation of embryonic gut showing the presence of a few differentiating enteric neurons (***A***, inset) revealed by anti-PGP9.5 antibody (***B***). Scale bars: 100 μm (***A***) and 50 μm (***B***).

We moved on to examine the effects of a reduction in the dosage of the *RET* gene because reduced RET expression is known to confer susceptibility to intestinal aganglionosis in both human and mice (Emison et al., 2005; Uesaka et al., 2008). We crossed *Ret^51/51^* or *Ret^51(C618F)/51(C618F^*^)^ mice to *Ret^EGFP/+^* mice in which one of the *Ret* alleles was replaced by the *Ret-EGFP* allele (*Ret* null). Consistent with previous observations that one allele of wild-type RET-expressing allele is sufficient for normal development of the ENS in mice, the gut was fully furnished with ENS meshwork in *Ret^51/EGFP^* mice (Fig. 3*A*,*B*, left). In a stark contrast, all of *Ret^51(C618F)/EGFP^* mice displayed intestinal aganglionosis (Fig. 3*B*). This result was surprising, as *Ret^51(C618F)/51(C618F^*^)^ mice display hyperganglionosis (Fig. 1*A*). Although the length of aganglionic gut was varied, in ∼62% of *Ret^51(C618F)/EGFP^* mice (29 out of 34 mice examined), the ENS was present only in the small intestine (Fig. 3*C*). Among these mice, eight mice (20% of all examined) displayed skip segment-type aganglionosis (Fig. 3*B*, third picture). This skip segment appears to be developed at least partially because of impaired migration of trans-mesenteric ENS progenitors, a cell population primarily contributing to colonic ENS (Nishiyama et al., 2012), because we occasionally found a limited number of enteric neurons scattered in the colon in some of *Ret^51(C618F)/EGFP^* embryos at E13.5, a period 2 d after trans-mesenteric migration is completed (Fig. 4). These data demonstrate that *RET51*(C618F) allele causes severe intestinal aganglionosis when the *RET* gene dosage is reduced to half.

### Premature neuronal differentiation impairs migration of ENS progenitors in *Ret^51(^*^C618F^*^)/EGFP^* embryos

**Figure 5.**
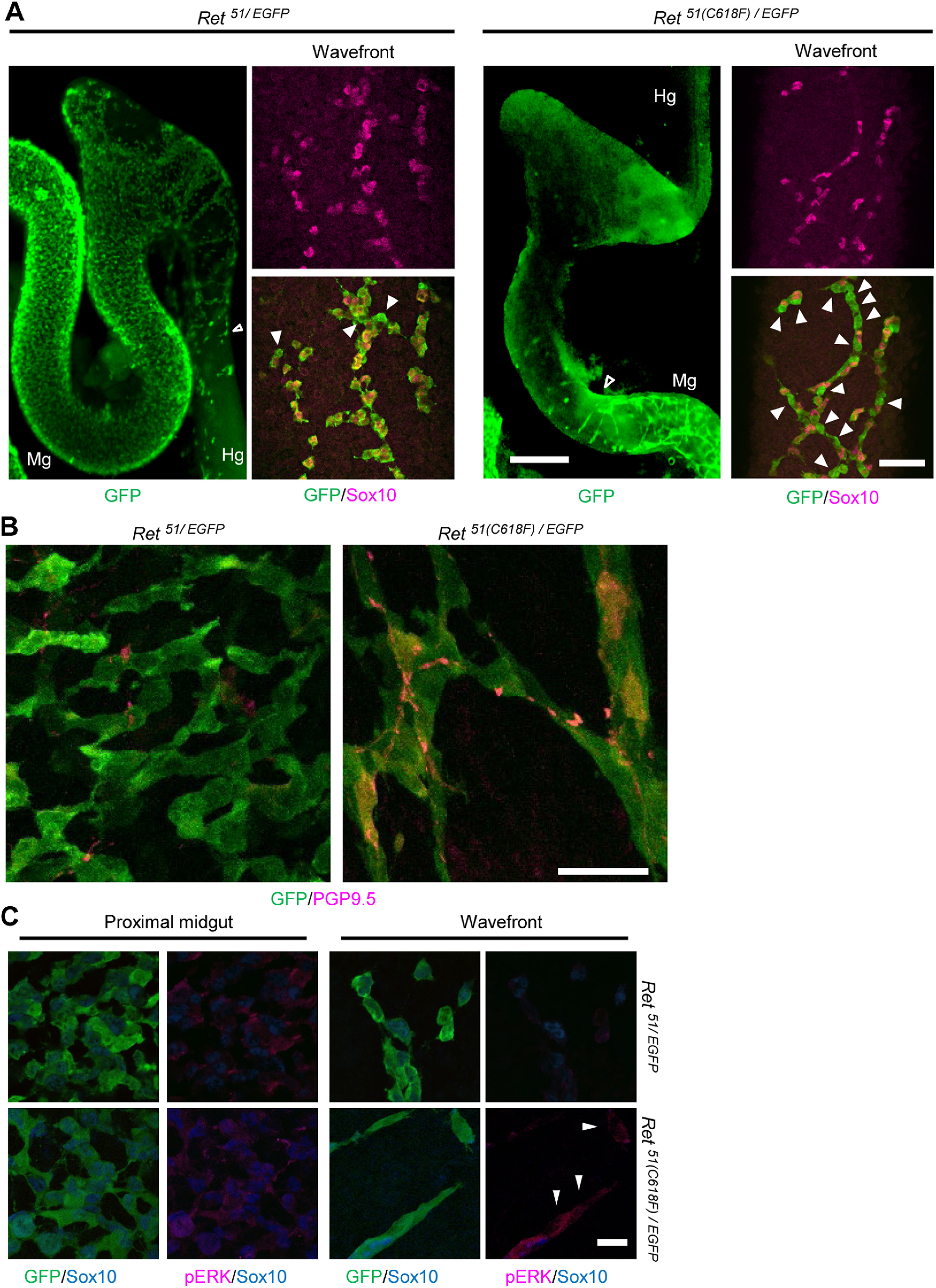
Reduced RET51(C618) expression leads to premature differentiation of ENS precursors at the migratory wavefront. ***A***, Whole-mount images of GFP-labeled cells in the gut from E12.5 *Ret^51/EGFP^* and *Ret^51(C618F)/EGFP^* embryos (left panels). GFP^+^ cells in the migratory wavefront were stained by anti-Sox10 (right panels), whereas Sox10-negative GFP^+^ cells (white arrowheads) were found at the delayed migratory wavefront (open arrowhead) of Ret*^51(C618F)/EGFP^* gut. ***B***, Whole-mount images of GFP-labeled cells stained with anti-PGP9.5 in E12.5 *Ret^51/EGFP^* and *Ret^51(C618F)/EGFP^* gut. PGP9.5-labeled GFP^+^ cells were detected at the delayed migratory wavefront of Ret*^51(C618F)/EGFP^* gut. ***C***, Immunohistochemical staining for GFP (green), Sox10 (blue), and activated ERK (pERK, magenta) in ENS cells of *Ret^51/EGFP^* and *Ret^51(C618F)/EGFP^* embryos at E12.5. In the migratory wavefront of *Ret^51(C618F)/EGFP^* embryos, pERK was mainly observed in GFP^+^ and Sox10^–^ differentiating neurons (white arrowheads). Hg, hindgut; Mg, midgut. Scale bars: 250 μm (***A***, left panel), 50 μm (***A***, right panel), 25 μm (***B***), and 20 μm (***C***).

To investigate the mechanism underlying the impaired ENS development in *Ret^51(C618F)/EGFP^* mice, we conducted whole-mount immunohistochemical analyses of embryonic gut (E12.5). In control (*Ret^51/EGFP^*) embryos, ENS progenitors at the migrating wavefront invaded the proximal colon and expressed both RET (revealed by GFP fluorescence) and Sox10 (Fig. 5*A*, left), indicating those cells are immature progenitors. Consistent with this expression pattern, none of the cells at the wavefront expressed PGP9.5 (Fig. 5*B*, left), a marker for neuronal differentiation. In contrast, in *Ret^51(C618F)/EGFP^*embryos, Sox10 expression was lost in many cells at the wavefront (Fig. 5*A*, right, arrowheads). Associated with this change, we found aberrant expression of PGP9.5 in ENS progenitors at the migratory wavefront, which was located primarily in the midgut (Fig. 5*B*, right). These results indicate that premature neuronal differentiation is induced in ENS progenitors at the migratory wavefront in *Ret^51(C618F)/EGFP^* embryos.

A previous study revealed that elevation of ERK activity is associated with induction of neuronal differentiation in ENS progenitors (Uesaka et al., 2013). We examined ERK activation by whole-mount staining of embryonic gut (E12.5) using anti-phospho-Erk (pErk) antibodies. In *Ret^51/EGFP^* embryos, pErk-positive ENS progenitors were abundant in proximal regions of the midgut, whereas such cells were almost undetectable at the wavefront region (Fig. 5*C*, right upper panels). In contrast, pErk-positive cells were frequently observed not only in the proximal midgut but also in the wavefront regions in *Ret^51(C618F)/EGFP^* embryos (Fig. 5*C*, left and right bottom panels). These data collectively indicate that single allele-only expression of RET51(C618F) causes premature enteric neuronal differentiation *in vivo*.

### GDNF-mediated activation of RET51(C618F) is responsible for premature neuronal differentiation of ENS progenitors

**Figure 6.**
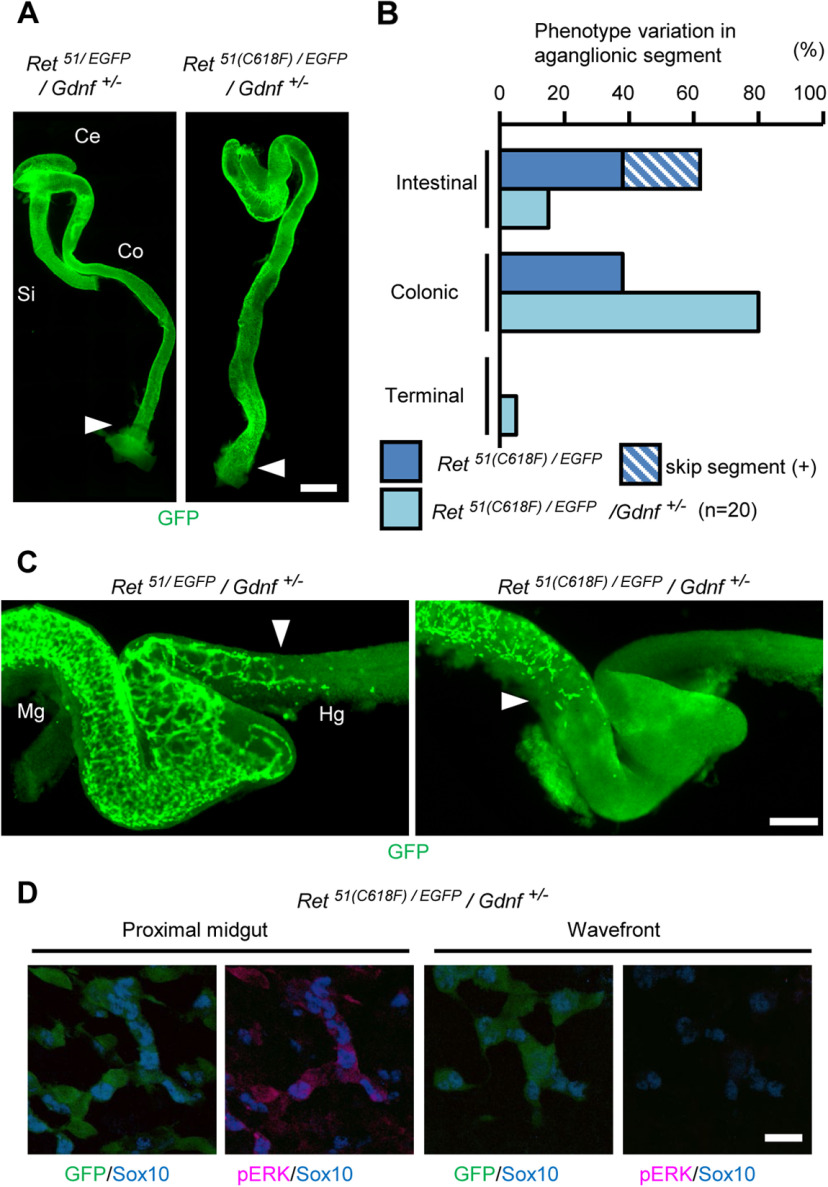
Reducing *Gdnf* gene dosage moderately rescues ENS phenotype of *Ret^51(C618F)/–^*mice. ***A***, Representative images of P0 *Ret^51/EGFP^/Gdnf^+/–^* and *Ret^51(C618F)/EGFP^/Gdnf^+/–^* large intestine showing complete colonization with GFP-positive enteric neurons. ***B***, Comparison of ENS wavefront location between *Ret^51(C618F)/EGFP^* and *Ret^51(C618F)/EGFP^/Gdnf^+/–^* mice at P0. Reduction of *Gdnf* gene dosage significantly ameliorated the severity of enteric aganglionosis (χ^2^ test, *p *<* *0.01). ***C***, Representative images of E12.5 *Ret^51/EGFP^/Gdnf^+/–^* and *Ret^51(C618F)/EGFP^/Gdnf^+/–^* gut displaying colonization by GFP-positive ENS precursors. White arrowheads indicate the location of ENS precursor wavefront. ***D***, Whole-mount GFP, Sox10, and pERK pathway stainings of ENS cells of *Ret^51(C618F)/EGFP^/Gdnf^+/–^* embryos at E12.5. Activation of ERK was not observed in ENS precursors at the migratory wavefront. Ce, cecum; Co, colon; Si, small intestine; Hg, hindgut; Mg, midgut. Scale bars: 1000 μm (***A***), 250 μm (***C***), and 20 μm (***D***).

Previous biochemical analyses revealed that RET51(C618F) responds to GDNF and displays enhanced phosphorylation *in vitro*. To investigate whether GDNF-induced stimulation of RET51(C618F) contributes to severe aganglionosis phenotype in *Ret^51(C618F)/+^* mice, we examined whether severity of the phenotype changes in *Ret^51(C618F)/EGFP^* mice on *Gdnf^+/–^*background. By whole-mount GFP staining of the neonatal gut (*n* = 20), we found that most of *Ret^51(C618F)/EGFP^*/*Gdnf^+/–^* mice (80%) displayed colonic aganglionosis (Fig. 6*A*), which stood in a sharp contrast to the ENS phenotype of *Ret^51(C618F)/EGFP^* that showed mostly extensive aganglionosis (aganglionic segment exceeding to the small intestine). χ^2^ test of independence confirmed the significant differences between *Ret^51(C618F)/EGFP^*/*Gdnf^+/–^* and *Ret^51(C618F)/EGFP^*/*Gdnf^+/+^* mice (*p *=* *0.005 < 0.01). Interestingly, in one case, the ENS was found fully developed up to the anal end (Fig. 6*B*). Moreover, skip segment-type aganglionosis, which was identified in 24% of *Ret^51(C618F)/-^*mice, was not detected in any of *Ret^51(C618F)/EGFP^*/*Gdnf^+/–^* mice. These results collectively indicate that reduction in GDNF levels exerts significant rescue effects on severe aganglionosis phenotype (Fig. 6*A*,*B*).

At E12.5, migration of ENS precursors was delayed in *Ret^51(C618F)/EGFP^/Gdnf^+/–^* embryos as compared with *Ret^51/EGFP^/Gdnf^+/–^* embryos (Fig. 6*C*). To examine the effect of the reduction of *Gdnf* gene dosage on intracellular signaling, whole-mount pERK staining was performed on the gut of *Ret^51(C618F)/EGFP^/Gdnf^+/–^* embryos (E12.5). Similar to wild-type or *Ret^51/EGFP^* embryos (Fig. 4*C*, top), ERK phosphorylation was undetectable at the wavefront regions of *Ret^51(C618F)/EGFP^/Gdnf^+/–^* embryos (Fig. 6*D*, right). These data reveal that GDNF-mediated activation of RET51(C618F) is responsible for aberrant phosphorylation of ERK in ENS precursors at the wavefront and causes intestinal aganglionosis in *Ret^51(C618F)/-^* embryos.

### An allelic loss of the *Ednrb* gene exacerbates the ENS phenotype of *Ret^51(C618F)/-^* mice

**Figure 7.**
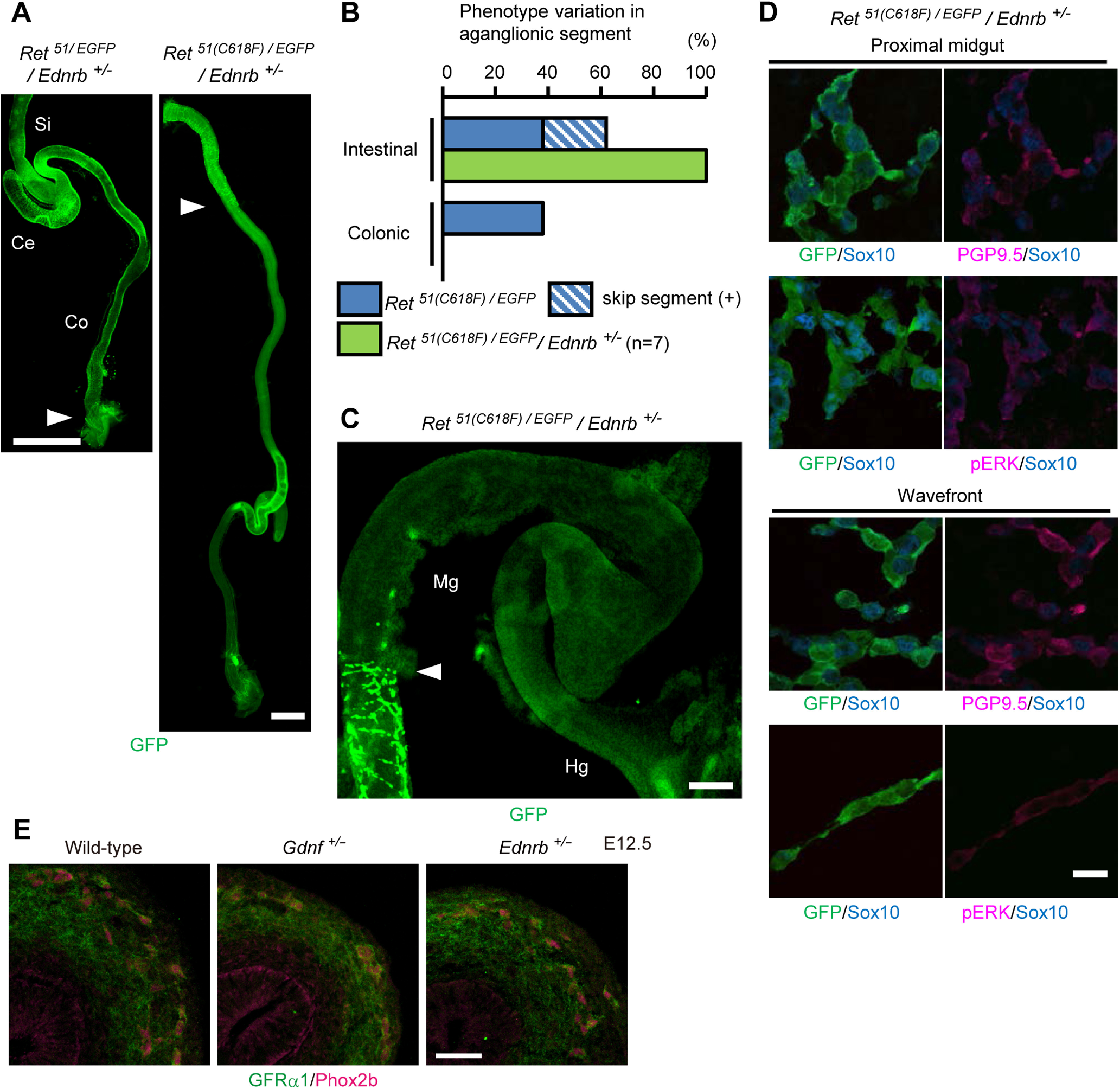
The severity of interruption of the ENS migration depends on the *Ednrb* gene dosage in *RET^51(C618F)/-^* mice. ***A***, Whole-mount GFP staining of P0 *Ret^51/EGFP^/Ednrb^+/−^* and *Ret^51(C618F)/EGFP^/Ednrb^+/−^* gut. White arrowheads indicate the location of wavefront of enteric neurons. ***B***, Comparison of location of ENS wavefront between *Ret^51(C618F)/EGFP^* and *Ret^51(C618F)/EGFP^/Ednrb^+/−^* mice at P0. Reduction of *Ednrb* gene dosage increased the severity of enteric aganglionosis of *Ret^51(C618F)/EGFP^*mice. ***C***, Whole-mount GFP staining of the enteric neurons in E12.5 *Ret^51(C618F)/EGFP^/Ednrb^+/−^* gut. White arrowhead indicates location of the wavefront of ENS precursors. ***D***, Whole-mount GFP, PGP9.5, Sox10, and pERK pathway stainings of ENS cells of *Ret^51(C618F)/EGFP^/Ednrb^+/−^* embryos at E12.5. Activation of ERK was observed in ENS precursors at the migratory wavefront. ***E***, Immunohistochemical detection of GFRα1 (green) in ENS progenitors (whose nuclei marked in magenta by anti-Phox2b antibody) of wild-type, *Gdnf^+/–^*, and *Ednrb^+/−^* embryos (E12.5). Ce, cecum; Co, colon; Si, small intestine; Hg, hindgut; Mg, midgut. Scale bars: 1 mm (***A***), 250 μm (***C***), and 20 μm (***D***), 50 μm (***E***).

Previous studies revealed a genetic interaction between the *Ret* and *Ednrb* genes in HSCR pathogenesis. Either *Ret* heterozygosity or the *Ednrb ls/ls* allele alone exerts no adverse effect on ENS development, but induces severe intestinal aganglionosis when combined (Carrasquillo et al., 2002). Ednrb signaling regulates multiple processes of ENS development including migration, proliferation and differentiation of ENS precursors (Barlow et al., 2003; Kruger et al., 2003). We examined a potential genetic interaction between the *Ret51(C618F)* allele and the *Ednrb* gene by crossing *Ret^51/51(C618F)^* mice to *Ednrb^+/−^*/*Ret^EGFP/+^* mice. We found that, in contrast to *Ret^51/EGFP^*/*Ednrb^+/−^* embryos, which displayed normal ENS development, *Ret^51(C618F)/EGFP^*/*Ednrb^+/−^* embryos exhibited severe intestinal aganglionosis in which the ENS is observed only in the proximal part of the small intestine (Fig. 7*A–C*). Immunohistochemical examination of ENS precursors revealed robust phosphorylation of ERK and loss of Sox10 expression at the wavefront regions (Fig. 7*D*). Thus, reduction of one copy of the *Ednrb* gene leads to exacerbation of the aganglionosis phenotype, which contrasted the ameliorating effect by the reduction of the *Gdnf* gene dosage. This difference is not caused by the differential expression levels of GFRα1, the cognate receptor for GDNF involved in HSCR pathogenesis (Lui et al., 2002), as it was expressed at comparable levels in *Gdnf^+/–^*, *Ednrb^+/−^*, and wild-type embryos (Fig. 7*E*). At any rate, these results reveal a clear genetic interaction between the *Ret51(C618F)* allele and the *Ednrb* gene and suggest that the Ednrb signaling functions to inhibit premature differentiation of ENS precursors in this context.

## Discussion

In this study, we have provided evidence that RET(C618F), a RET-activating mutant, causes intestinal aganglionosis when the *Ret* gene copy number is reduced to half, which is contrary to the current consensus that enteric aganglionosis is caused by inactivating RET mutations. This unexpected finding provides novel insights into mechanisms underlying the development of the ENS by RET/GDNF signaling and the pathogenesis of HSCR.

The involvement of RET activating mutations in HSCR was first described in co-segregation of MEN2A/FMTC and HSCR in a fraction of families. These patients carry missense mutations in the *RET* gene, which substitutes arginine or serine for a cysteine residue at position 618 or 620. These RET mutants display ligand-independent constitutive activation because of intermolecular di-sulfide-linked dimerization (Santoro et al., 1995) and simultaneously loses cell surface expression (Asai et al., 1995). The former property contributes to neoplastic pathology including MTC and pheochromocytoma, whereas the latter contributes to impaired development of the ENS (HSCR). Therefore, in the context of ENS development, these mutants (C618R, C618S and C620R) behave as RET-inactivating mutants (Mulligan et al., 1994; Borst et al., 1995). Animal studies support this notion, as mice harboring RET(C620R) mutation in a homozygous fashion display kidney agenesis and intestinal aganglionosis, a phenotype identical to that of *Ret*-deficient mice (Carniti et al., 2006; Yin et al., 2007). RET(C618F) examined in this study exhibited distinct properties. Unlike other C618 or C620 mutants, RET(C618F) is expressed on the cell surface (Okamoto et al., 2019). Although phosphorylation levels of RET(C618F) are slightly higher than those of wild-type RET, GDNF stimulation further enhances phosphorylation of RET(C618F) (Okamoto et al., 2019). Thus, RET(C618F) is a GDNF-responsive RET-activating mutant. Consistent with these biochemical properties, *Ret^51(C618F)/51(C618F^*^)^ mice had increased numbers of enteric neurons because of enhanced proliferation of ENS precursors. Surprisingly, despite the activating nature of RET51(C618F), *Ret^51(C618F)/-^* mice displayed severe intestinal aganglionosis, in sharp contrast to *Ret^RET51/-^* mice (control), which exhibited no ENS deficit. This study provides evidence, for the first time to our knowledge, that RET-activating mutations can cause intestinal aganglionosis when coupled with a reduction in the *Ret* gene dosage.

It is important to note that, the *RET C618F* allele displays genetic interaction with the *Ednrb* gene, which is known as a modifier gene for HSCR carrying mutations in the *RET* gene. Our findings suggest a novel pathogenetic mechanisms of HSCR by revealing how reduced RET expression affects ENS development and confers susceptibility to HSCR (Emison et al., 2005). It is also important to note that many of *Ret^51(C618F)/-^* mice displayed skip-segment aganglionosis. *Ret^51(C618F)/-^* mice thus serve as the first valuable platform to investigate the molecular and cellular mechanisms underlying this mysterious condition.

Histologic examination of *Ret^51(C618F)/-^* embryos revealed that premature neuronal differentiation of ENS precursors is likely to be the cause of the intestinal aganglionosis. Exacerbation of the aganglionic phenotype by the reduction of the *Ednrb* gene supports this possibility because endothelin-3/Ednrb signaling prevents premature neuronal differentiation (Wu et al., 1999). The aganglionic phenotype of *Ret^51(C618F)/-^* embryos stands in sharp contrast to that of *Ret^51(C618F)/51(C618F^*^)^ embryos, in which ENS precursors underwent proliferation rather than differentiation. Although the exact mechanism by which ENS precursors adopt to a different cell fate (proliferation or differentiation) is unknown, it may involve regulation of Erk phosphorylation. In PC12 cells, EGF treatment enhances cell proliferation, while FGF treatment induces neuronal differentiation. This difference in cell fate is tightly associated with the levels and kinetics of Erk phosphorylation. EGF evokes a rapid surge and subsequent abrupt quenching of Erk phosphorylation, whereas FGF induces long-lasting and moderate levels of Erk phosphorylation (Qiu and Green, 1991; Traverse et al., 1992; Nguyen et al., 1993). Interestingly, Erk phosphorylation-associated cell fate determination in ENS precursors was reported previously (Natarajan et al., 2002; Asai et al., 2006; Goto et al., 2013) . We can therefore assume that Erk activity is differentially regulated in ENS precursors between *Ret^51(C618F)/51(C618F^*^)^ and *Ret^51(C618F)/-^* embryos. We tried to examine this possibility by culturing ENS precursors and conducting biochemical analyses. Unfortunately, however, *Ret^51(C618F)/-^* ENS precursors displayed a tendency to differentiate *in vitro*, and we were unable to obtain reliable data. To understand the mechanisms underlying the intestinal aganglionosis in *Ret^51(C618F)/-^* mice, we also have to understand the biochemical properties of RET51(C618F) in more detail. Although RET51(C618F) is expressed on cell surface, it is also detected in the cytoplasm (Okamoto et al., 2019). The latter likely reflects localization in ER, which is commonly observed in all MEN-associated RET mutant proteins (Wagner et al., 2012). Thus, RET51(C618F) has combined properties of wild-type RET and MEN-associated RET mutants. Even on the cell surface, it is unknown whether RET51(C618F) behaves as wild-type RET. For instance, on GDNF binding to GFRα receptors, wild-type RET protein gets recruited to the raft and phosphorylated, which provides a platform to activate Src and Akt-PI3 kinase efficiently. It is currently unknown how RET51(C618F) is localized and activates intracellular signaling molecules on the cell surface. In these respects, RET51(C618F) may not reflect enhanced activity of purely wild-type RET. RET51(C618F) has unique biochemical properties among MEN-associated RET mutants, which suggest that all of RET-activating mutations do not necessarily cause the intestinal aganglionosis by *RET* gene dosage reduction.

Amelioration of the phenotype in *Ret^51(C618F)/-^* embryos by the *Gdnf* gene reduction indicates that GDNF-mediated activation of RET51(C618F) is responsible for the severe aganglionic phenotype. Although NRTN also activates RET in developing ENS (Heuckeroth et al., 1998), contribution of NRTN-mediated RET(C618F) activation to the aganglionic phenotype is unlikely because expression of GFRα2, the cognate receptor for NRTN, occurs later than a period when the aganlionic phenotype in *Ret^51(C618F)/-^* embryos becomes obvious. It is important to note that, in normal ENS development, enteric neuron numbers are determined primarily by the levels of GDNF signaling. Mice heterozygous for the GDNF-deficient allele (*Gdnf^+/–^* mice) display reduced numbers of enteric neurons (Gianino et al., 2003). In contrast, reduction of Sprouty2, an inhibitor of Erk phosphorylation downstream of RET/GDNF signaling, leads to hyperganglionosis of the gut (Taketomi et al., 2005). This hyperganglionosis phenotype is suppressed on *Gdnf^+/–^* background. Evidence also suggests that RET expression is regulated by RET activity induced by GDNF (Oppenheim et al., 2000). Taken together, both signaling and expression of RET are exquisitely controlled by the availability of GDNF, Sprouty2 and phosphor-Erk. Even a slight disturbance (both upregulation and downregulation) of RET signaling can abrogate ENS development (Nagy et al., 2020). Understanding the development and developmental disorders of the ENS requires the elucidation of interactions among these molecules.

## Synthesis

Reviewing Editor: Eran Perlson, Tel Aviv University Decisions are customarily a result of the Reviewing Editor and the peer reviewers coming together and discussing their recommendations until a consensus is reached. When revisions are invited, a fact-based synthesis statement explaining their decision and outlining what is needed to prepare a revision will be listed below. The following reviewer(s) agreed to reveal their identity: Michael Gershon.

In this study the authors of the paper "Increased RET activity coupled with a reduction in the RET gene dosage causes intestinal aganglionosis in mice" use advanced transgenic technologies to study the effect of RET51(C618F) mutant, an activating mutation identified in MTC patients, in the development of ENS. The study provides the first evidence that RET-activating mutations can actually cause intestinal aganglionosis when they are coupled with a reduction in the dosage of the Ret gene. It also produces skip lesions and verifies that premature differentiation of migrating crest-derived cells as neurons leads to terminal aganglionosis of the bowel. It is highly interesting and original idea that is truly against the common belief that HSCR and MTC are induced by inactivating and activating RET mutations, respectively.

In earlier study it was shown that Ret51(C618F)/51(C618F) mice displayed hyperganglionosis of the ENS and C-cell hyperplasia. Importantly, it was shown that Ret51(C618F) is slightly more phosphorylated than wt RET and that it can be further phosphorylated by GDNF.

Now authors show that Ret51(C618F)/- mice have severe intestinal aganglionosis due to premature neuronal differentiation. Then crossing these mice with GDNF heterozygotes ameliorated the ENS phenotype of Ret51(C618F)/- mice. This result clearly demonstrates that it is the GDNF-mediated activation of RET51(C618F) that is responsible for severe aganglionic phenotype. Finally, they show that RET51(C618F) has genetic interaction with Ednrb gene, which is well known as one of modifier genes for HSCR.

This is an elegant manuscript well written and controlled. The genetic analysis has been done very well. This study provides the first evidence that RET-activating mutations can actually cause intestinal aganglionosis when they are coupled with a reduction in the dosage of the Ret gene. It is also interesting that the RET C618F allele, which the authors study interacts with Ednrb, which is a modifier gene for RET mutations associated with the Hirschsprung (HSCR) phenotype. The paper makes a strong case for premature differentiation of neurons as a cause of aganglionosis of the terminal bowel.

In order to strengthen the conclusions some additional information is needed:

1. It is clear from the previous study that in RET51(C618F) mutant RET is phosphorylated both on the cell surface and also in the ER (the lower band in Western blot). GDNF is exclusively activating RET on the cell surface in the presence of GFRa1. It is also now well demonstrated HSCR that significant reduction in the level of GFRa1 can cause HSCR. Authors also show that reduction of one copy of the Ednrb gene leads to exacerbation of the aganglionosis phenotype revealing a clear genetic interaction between the Ret51(C618F) allele and the Ednrb gene. The question arises, whether GFRa1 can play a role here? Authors should analyse the level of GFRa1 (both total and cell surface) in ENS in mice with GDNF and Ednrb heterozygosity.

2. Since GDNF signalling seems to play a key role here it is possible that RET signalling in the ER and on the cell surface (where GDNF act) differ? Authors should at least discuss potential role of Src activation that mainly occurs in the rafts, as well as Akt-PI-3 kinase signalling.

3. Many of the HSCR mutation have defect in intracellular trafficking and they do not reach the cell surface. Is there any difference in the RET cell surface location RET51(C618F) mutant?

In the Summary authors write that GDNF is the RET ligand. This is not entirely correct. It is the GFRa1-RET ligand.

In the introduction authors should also discuss MEN2B mutation and its signalling, because this is known to be independent of GDNF.

Authors should also discuss the role of GFRa1 and NRTN in the HSCR and mention that MEN2 mutants also signal inside the cells.

4. The authors should cite an earlier study that also presented evidence for premature differentiation of neurons as a cause of terminal gut aganglionosis, albeit under different genetic circumstances; for instance, Wu et al suggested in 1999 that that endothelin-3/endothelin B signaling normally prevents the premature differentiation of crest-derived precursors migrating to and within the fetal bowel, enabling the precursor population to persist long enough to finish colonizing the bowel (Wu JJ, Chen J-X, Rothman TP, Gershon MD (1999) Inhibition of in vitro enteric neuronal development by endothelin-3: mediation by endothelin B receptors. Development 126:1161-1173). This is a different mechanism than that discussed by the authors; however, the mechanism is similar and Ednrb is involved.

5. It is also amazing that many of the affected mice display skip lesions. The authors note this and blandly state that their model might be interesting for future studies of that mysterious phenomenon. I think the authors should do a little better than that and actually suggest a proposed mechanism. It is not clear from the studies that the authors describe.

In summary, this is a beautiful study that needs some modification and clarifications to be a major advance in knowledge of enteric neuronal development and the pathogenesis of HSCR

## Author Response

Dear Dr. Perlson:

Reviewing Editor

eNeuro

Please find the revised version of manuscript eN-TNWR-0534-20 "Increased RET activity

coupled with a reduction in the RET gene dosage causes intestinal aganglionosis in mice" that we

have submitted for publication in eNeuro. We appreciate the positive comments from the

reviewers regarding our manuscript. We are especially pleased that they have found our study

elegant and thorough. We have attempted to address all of the reviwers’ comments, which we

believe have made the revised manuscript stronger and more complete. Our point-by-point

response to reviewers’ comments follow.

[Comment 1] It is clear from the previous study that in RET51(C618F) mutant RET is

phosphorylated both on the cell surface and also in the ER (the lower band in Western blot).

GDNF is exclusively activating RET on the cell surface in the presence of GFRa1. It is also now

well demonstrated HSCR that significant reduction in the level of GFRa1 can cause HSCR.

Authors also show that reduction of one copy of the Ednrb gene leads to exacerbation of the

aganglionosis phenotype revealing a clear genetic interaction between the Ret51(C618F) allele

and the Ednrb gene. The question arises, whether GFRa1 can play a role here? Authors should

analyse the level of GFRa1 (both total and cell surface) in ENS in mice with GDNF and Ednrb

heterozygosity.

[Response to Comment 1] We thank the reviewer for pointing this out. We have added sentences

to explain subcellular localization of RET51(C618F) in Discussion (lines 350-356). We also

examined the expression of GFRa1 in GDNF+/- and Ednrb+/- mice, which showed no discernible

differences as compared to wild type mice (Results: lines 283-287). These results were included

in Figure 6E.

[Comment 2] Since GDNF signaling seems to play a key role here it is possible that RET signaling

in the ER and on the cell surface (where GDNF act) differ? Authors should at least discuss

potential role of Src activation that mainly occurs in the rafts, as well as Akt-PI-3 kinase

signaling.

[Response to Comment 2] We have added sentences to explain the potential differences in RET

signaling between ER and cell surface (Discussion: lines 350-355), and ones to explain RET

signaling in the rafts (Discussion: lines 355-364).

[Comments 3] In the Summary authors write that GDNF is the RET ligand. This is not entirely

correct. It is the GFRa1-RET ligand. In the introduction authors should also discuss MEN2B

mutation and its signaling, because this is known to be independent of GDNF. Authors should

also discuss the role of GFRa1 and NRTN in the HSCR and mention that MEN2 mutants also

signal inside the cells.

[Response to Comments 3] We thank this reviewer’s comment. We agree that GDNF is the

GFRa1-RET ligand. However, due to the word number limitation, we have no space to explain

GFRa receptors in the Abstract. We therefore described GDNF as ‘a RET-activating neurotrophic factor’ (line 10). We have also added sentences to explain that MEN2-asscociated RET mutant

can be activated in a ligand-independent manner in the introduction (line 60). We have also

discussed the role of GFRa1 and NRTN in Discussion (lines 367-372).

[Comment 4] The authors should cite an earlier study that also presented evidence for premature

differentiation of neurons as a cause of terminal gut aganglionosis, albeit under different genetic

circumstances; for instance, Wu et al suggested in 1999 that that endothelin-3/endothelin B

signaling normally prevents the premature differentiation of crest-derived precursors migrating

to and within the fetal bowel, enabling the precursor population to persist long enough to finish

colonizing the bowel (Wu JJ, Chen J-X, Rothman TP, Gershon MD (1999) Inhibition of in vitro

enteric neuronal development by endothelin-3: mediation by endothelin B receptors.

Development 126:1161-1173). This is a different mechanism than that discussed by the authors;

however, the mechanism is similar and Ednrb is involved.

[Response to Comment 4] The authors appreciate the reviewer’s suggestion. We have added the

paper in references (lines 332-335).

[Comment 5] It is also amazing that many of the affected mice display skip lesions. The authors

note this and blandly state that their model might be interesting for future studies of that

mysterious phenomenon. I think the authors should do a little better than that and actually suggest

a proposed mechanism. It is not clear from the studies that the authors describe.

[Response to Comment 5] Although we have not thoroughly examined the mechanism underlying

the formation of skip lesions, we have obtained some evidence suggesting that impaired migration

of trans-mesenteric ENCCs contributes to skip lesions. We have added some of those data in

Figure 4 and explained the potential involvement of trans-mesenteric ENCCs (lines 209-214).

Thank you very much again for considering our work for publication in eNeuro.

Sincerely,

Hideki Enomoto
